# Functional reconstruction after subtotal glossectomy in the surgical treatment of an uncommon and aggressive neoplasm in this location: Primary malignant melanoma in the base of the tongue

**DOI:** 10.4317/jced.51606

**Published:** 2014-10-01

**Authors:** Isidoro Rubio-Correa, Damián Manzano-Solo-de-Zaldívar, Manuel Moreno-Sánchez, Cristina Hernández-Vila, Francisco-Alejandro Ramírez-Pérez, David González-Ballester, Luis Ruíz-Laza, Raúl González-García, Florencio Monje-Gil

**Affiliations:** 1 Resident Surgeon. University Hospital Infanta Cristina, Department of Oral and Maxillofacial Surgery, Badajoz, Spain; 2 Attending Surgeon. University Hospital Infanta Cristina, Department of Oral and Maxillofacial Surgery, Badajoz, Spain; 3 Head of Department of Oral and Maxillofacial. University Hospital Infanta Cristina, Badajoz, Spain

## Abstract

Primary malignant melanoma of the oral cavity is a rare neoplasm, especially on the tongue. We report a case of mucosal melanoma at the base of the tongue, an extremely rare location (only about 30 cases have been reported in literature). The extension study doesn´t revealed distant metastatic lesions. The patient was treated by subtotal glossectomy and bilateral functional neck dissection. Tongue is one of the most difficult structures to reconstruct, because of their central role in phonation, swallowing and airway protection. The defect was reconstructed with anterolateral thigh free flap. Surgical treatment was supplemented with adjuvant immunotherapy. The post-operative period was uneventful. At present, 24 months after surgery, patient is asymptomatic, there isn´t evidence of recurrence of melanoma and he hasn´t any difficulty in swallowing or phonation.

** Key words:**Malignant mucosal melanoma, anterolateral thigh free flap, phonation, swallowing.

## Introduction

Primary malignant melanoma in oral cavity is rare neoplasm. It represents approximately 1.7 % of all melanomas and 6.3% of head and neck melanoma (1). It occurs with equal frequency in the male and female and is seen most commonly in the Caucasian). In the oral cavity, they appear with more frequency in maxillary gingiva, palate mucosa and lips. However, primary malignant melanoma is specifically uncommon in the tongue. In the literature, only about 30 cases have been reported. We describe a case of malignant melanoma primarily originating from the base of the tongue, treated successfully with resection. The defect was reconstructed with fasciocutaneous anterolateral thigh flap.

## Case Report

**Figure 1:**
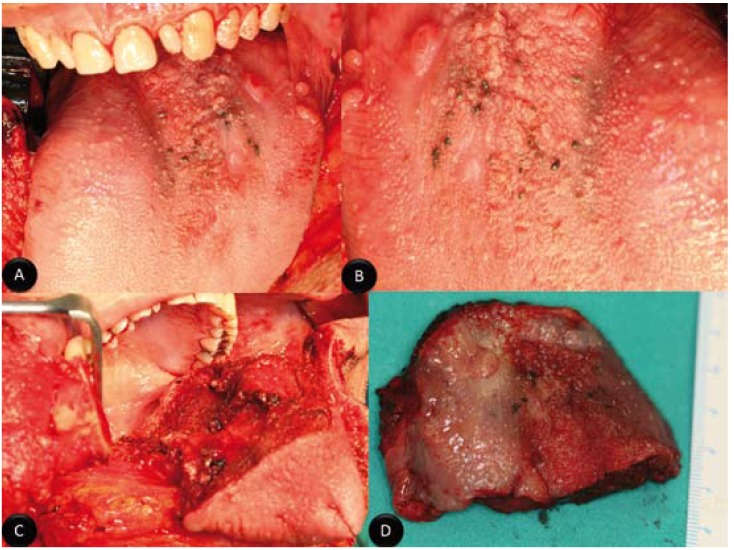
Appeaence of lesion. A) Photograph showing a pigmented and diffuse mass measuring approximately 3 X 3 cm in size on the base of the tongue; B) Photograph showing augmented image of lesion. Intraoperative images; C) Intraoperative photograph showing the subtotal glossectomy via mandibular swing procedure; D) Surgical specimen.

**Figure 2:**
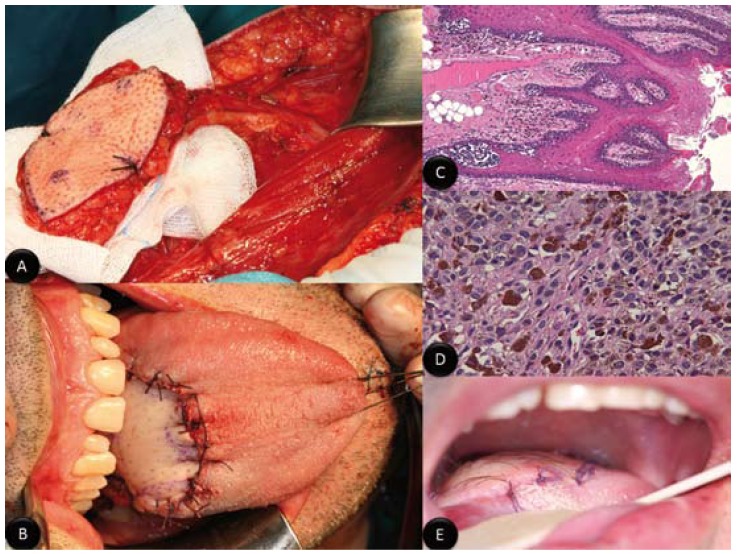
A) Photograph showing fasciocutaneous anterolateral thigh flap used in reconstruction of the defect; B) Appearance of neotongue; C) Photomicrograph showing widespread atypical melanocytes at the mucosal–submucosal junction, rare Pagetoid upward migration of these cells and underlying invasive melanoma (haematoxylin and eosin, original magnification × 100); D) Photomicrograph showing considerable pleomorphism of the nuclei and several mitotic figures (haematoxylin and eosin, original magnification × 400); E) Photograph showing the aspect of anterolateral thigh flap only one month after surgery

A 51-year-old man with a clinical history of esophageal hiatal hernia, gastroesophageal reflux, hepatitis A in childhood and appendectomy was referred to our department due to an asymptomatic pigmented lesion in the base of the tongue. On examination, a black, pigmented and diffuse mass measuring approximately 3 X 3 cm in size was found on the base of the tongue (Fig. 1). These lesions were asymptomatic. There weren´t trismus, dysphagia or odynophagia. The mobility of the tongue wasn´t disturbed. There was no significant cervical lymphadenopathy. Biopsy of the tongue lesion revealed a histopathology consistent with primary malignant melanoma. The extension study included cervicofacial, thoracoabdominal and pelvic Computed Tomography (Whole-body-CT) and Positron Emission Tomography (PET scan) The whole-body-TC was absolutely normal, while PET-CT demonstrated a hypermetabolic focus at the base of the tongue, but it discarded distant metastatic lesions. The case was discussed in the tumor board, and the decision was to perform surgical treatment. Then, under general anesthesia, the patient was placed in the supine position and a tracheotomy was made. The resection of base of tongue lesions was performed with safety margins of 2 cm. So, a subtotal glossectomy via mandibular swing procedure was made (Fig. 1). It retained the left lingual artery to ensure vascularization and thus the viability of the remaining tongue. Tongue defect was reconstructed by a 6 x 6 cm fasciocutaneous anterolateral thigh flap nourished by a septocutaneous perforator of the descending branch of the lateral circumflex femoral artery (Fig. 2). It was possible primary closure of the donor site. The recipient vessels were the superior thyroid artery and right lingual vein. The post-operative period was eight days, and it was uneventful. Definitive hystopathologic examination demonstrated a primary malignant mucosal melanoma with free of tumor excision margins. It was classified in T3 NO MO staging (Fig. 2). The case was again discussed in the tumor board, and the decision was to perform adjuvant immunotherapy. So, two weeks after surgery, the patient began immunotherapy treatment with interferon alfa-2b at high doses according to the Kirkwood scheme (induction: interferon-α2b: 20 million/m2, i.v., 5 days a week for four weeks; maintenance: interferon-α2b: 10 million/m2, s.c., three times a week for 48 weeks). Immunotherapy was well tolerated. At present, 24 months after surgery, patient is asymptomatic, there isn´t evidence of recurrence of melanoma and he hasn´t any difficulty in swallowing or phonation (Fig. 2).

## Discussion

According Gutman *et al* (2), the presence of melanocytes in the mucosal membrane of respiratory, digestive and urogenital tracts explains the occurrence of malignant melanoma in these locations. In the literature review, we found that most of case series of malignant melanoma in oral cavity, for example in Moore and Martin (1) or Rapini *et al* (3) series, include the tongue as possible location. However, the base of the tongue is an extremely rare location (4). The tongue is one of the most difficult structures of the oral cavity to reconstruct because of its central role in swallowing, phonation and airway protection (5). Reconstructive options of the tongue include two categories: to maintain mobility or to provide bulk (6). After glossectomy with 30 to 50 percent preservation of the original musculature, maintaining the mobility of the remaining tongue by a thin, pliable flap is preferred (7). This can be achieved by infrahyoid myofascial, medial sural artery perforator flap, radial forearm free flap, anterolateral thigh and ulnar forearm flap (7). When the post-resectional volume is less than 30 percent of the original tongue, the reconstruction shifts to restoration of bulk to facilitate swallowing by providing contact of the neotongue with the palate. Flaps providing bulk include the rectus abdominis myocutaneous free flap, latissimus dorsi myocutaneous free flap, pectoralis major musculocutaneous flap and trapezius island flap (7). Ante-rolateral thigh flap has emerged in recent decades as a popular option for head and neck reconstruction because of its reliability, long pedicle, and good donor-site morbidity (6). Because of its versatility, it has been used for providing bulk and for ensuring mobility (6). Another crucial aspect in melanoma management is the role of adjuvant treatment, due its poor prognosis. The main indication of radiotherapeutic treatment would be locoregional control of the disease. Respect adjuvant immunotherapy, the only officially accepted treatment is interferon alfa-2b according to the Kirwood scheme (8). However, its use is controversial because this therapy only has proved to be useful in the increase of the disease-free period, but it not change the overall survival. In conclusion, melanomas in the tongue are rare neoplasms. Treatment of mucosal melanomas should be aggressive, due to their poor prognosis. Nevertheless, it´s possible the functional reconstructive treatment that preserves swallowing or phonation. For this, microsurgical reconstruction with anterolateral thigh free flap is an adequate option.
